# Serum fetuin-A and RANKL levels in patients with early stage breast cancer

**DOI:** 10.5937/jomb0-37386

**Published:** 2023-03-15

**Authors:** Cigdem Usul Afsar, Hale Aral, Orçun Can, Trabulus Didem Can, Didem Karacetin, Nazlı Mehmet Ali, Gursu Rıza Umar, Senem Karabulut

**Affiliations:** 1 Istinye University Medical Faculty, Liv Vadi Istanbul Hospital, Department of Internal Medicine and Medical Oncology, Istanbul, Turkey; 2 University of Health Sciences, Istanbul Training and Research Hospital, Department of Medical Biochemistry, Istanbul, Turkey; 3 Goztepe Medical Park Hospital, Department of General Surgery, Istanbul, Turkey; 4 University of Health Sciences, Cam and Sakura Education and Research Hospital, Department of Radiation Oncology, Istanbul, Turkey; 5 University of Health Sciences, Cam and Sakura Education and Research Hospital, Department of Radiology, Istanbul, Turkey; 6 Acıbadem Bakirkoy Hospital, Department of Medical Oncology, Istanbul, Turkey; 7 Istanbul University, Institute of Oncology, Departent of Medical Oncology, Turkey

**Keywords:** breast cancer, fetuin-A, RANKL, serum, rak dojke, fetuin-A, RANKL, serum

## Abstract

**Background:**

Breast cancer (BC) is the primary cause of mortality due to cancer in females around the world. Fetuin-A is known to increase metastases over signals and peroxisomes related with growing. Receptor activator of nuclear factor-kB ligand (RANKL) takes part in cell adhesion, and RANKL inhibition is used in the management of cancer. We aimed to examine the relationship between serum fetuin-A, RANKL levels, other laboratory parameters and clinical findings in women diagnosed with early stage BC, in our population.

**Methods:**

Women having early stage BC (n=117) met our study inclusion criteria as they had no any anti-cancer therapy before. Thirty-seven healthy women controls were also confirmed with breast examination and ultrasonography and/or mammography according to their ages. Serum samples were stored at -80°C and analysed via ELISA.

**Results:**

Median age of the patients was 53 (range: 57-86) while it was 47 (range: 23-74) in the healthy group. Patients had lower high-density lipoprotein levels (p=0.002) and higher neutrophil counts (p=0.014). Fetuin-A and RANKL levels did not differ between the groups (p=0.116 and p=0.439, respectively) but RANKL leves were found to be lower in the favorable histological subtypes (p=0.04).

**Conclusions:**

In this study, we found no correlation between serum fetuin-A levels and clinical findings in patients diagnosed with early stage BC. However, RANKL levels are found to be lower in subgroups with favorable histopathologic subtypes such as tubular, papillary and mucinous BC and there was statistically significant difference.

## Abstract (sr)

**Uvod:**

Rak dojke (BC) je primarni uzrok smrtnosti od raka kod žena širom sveta. Poznato je da fetuin-A povećava metastaze u odnosu na signale i peroksizome povezane sa rastom. Receptorski aktivator liganda nuklearnog faktora-kB (RANKL) učestvuje u ćelijskoj adheziji, a RANKL inhibicija se koristi u lečenju kancera. Cilj NAM je bio da ispitamo odnos između serumskog fetuina-A, nivoa RANKL, drugih laboratorijskih parametara i kliničkih nalaza kod žena sa dijagnozom ranog stadijuma BC, u našoj populaciji.

**Metode:**

Žene sa ranim stadijumom BC (n=117) ispunjavale su kriterijume za uključivanje u našu studiju pošto ranije nisu imale nikakvu terapiju protiv raka. Kontrola 37 zdravih žena je takođe potvrđena pregledom dojki i ultrasonografijom i/ili mamografijom u skladu sa njihovim godinama. Uzorci seruma su čuvani na -80 °C i analizirani pomoću ELISA.

**Rezultati:**

Srednja starost pacijenata bila je 53 godine (raspon: 57-86) dok je u zdravoj grupi bila 47 (raspon: 23-74). Pacijenti su imali niži nivo lipoproteina visoke gustine (p=0,002) i veći broj neutrofila (p=0,014). Nivoi fetuina-A i RANKL nisu se razlikovali između grupa (p=0,116 i p=0,439, respektivno), ali je utvrđeno da su nivoi RANKL niži kod povoljnih histoloških podtipova (p=0,04).

**Zaključak:**

U ovoj studiji nismo pronašli korelaciju između nivoa fetuina-A u serumu i kliničkih nalaza kod pacijenata sa dijagnozom ranog stadijuma BC. Međutim, utvrđeno je da su nivoi RANKL niži u podgrupama sa povoljnim histopatološkim podtipovima kao što su tubularni, papilarni i mucinozni BC i postojala je statistički značajna razlika.

## Introduction

Breast cancer (BC) is the second most frequently diagnosed malignancy just behind lung cancer and also the primary cause of mortality due to cancer in female around the world. Over 1.5 million women (25% of all women with cancer) are diagnosed with BC every year throughout the world [1]. Today, the total number of BC patients have increased in response to exposure to several risk factors such as abnormal levels of estrogen, smoking, alcohol and obesity [2]. Nevertheless, we know that early BC detection could reduce BC death rates significantly in the long-term [3].

Therefore, specific screening methods with the use of the correct biomarkers are important in the detection of early stage BC [4]. Specially, blood, saliva, and urine were considered as ideal origin in which to assign the presence of cancer biomarkers such as annexin, peroxiredoxin and calreticulin [5].

Fetuin-A, also called Alpha 2-Heremans Schmid Glycoprotein (AHSG), is a serum glycoprotein synthesized by the liver and secreted into the blood stream [6]. Its founded principal role is the inhibition of ectopic calcification, but mounting evidence suggests that it is a multifunctional protein capable of modulating a number of critical signaling pathways and it has roles in disease processes such as diabetes mellitus and kidney disease [7]. In particular, high fetuin-A concentrations are found to be associated with atherogenic lipid profile and metabolic syndrome, low fetuin-A levels are related to vascular calcifications and inflammation [8]. In addition, there are few suggesting increased fetuin-A level may be a new serum biomarker in early BC [9].

Receptor activator of nuclear factor-kappa B ligand (RANKL) appertain to tumour necrosis factor superfamily which is a group of proteins that act as bidirectional signalling molecules. RANKL with osteocyte origin induces bone destruction by stimulating osteoclasts, while RANKL released from osteoblasts functions in the reverse effect [10]. At the same time, available evidence suggests that the RANKL signaling system is associated with in almost all steps in BC development, from primary oncogenesis to the establishment of secondary tumors in the bone [10].

The potential role of tumor markers is to improve early cancer determination and they significantly provide an unmatched opportunity to understand the disease’s biology, improve diagnosis and enhance treatment. In the present study; we aimed to investigate the the serum levels of fetuin-A and RANKL and their relationship with clinical parameters in patients with early stage BC.

## Materials and methods

### Demographic features of patients

A total of 117 female patients with early stage BC between the ages of 27–86 years (median: 53) were included in the study. Our control group was 37 healthy women between the ages of 23–74 (median: 47) years. BC was diagnosed according to the ultrasonography and histopathologic findings of the patients. Healthy controls were also confirmed with breast examination and ultrasonography and/or mammography according to their ages. The patients were asked for their medical history (type 2 diabetes, hypertension, smoking and medications) and measured for their glomerular filtration rate (GFR) and other biochemical parameters. Exclusion criteria included trauma history, major surgical history, chronic kidney disease with a creatinine clearance under 15 mL/min, hepatitis (alcoholic, toxic hepatitis, chronic autoimmune), fatty liver, alcoholic and primer biliary cirrhosis, chronic inflammatory disease, acute infection, known lung or liver disease, known rheumatic heart valve disease, congenital heart disease including bicuspid aorta, dilated cardiomyopathy and known osteoporosis.

### Serum preparation

Blood was drawn after 12 hours of fasting in the morning. Serum was obtained after at least 30 minutes of clotting by centrifugation at 2500xg for 15 minutes. Serum was stored at –80°C until assayed. All icteric or haemolytic blood samples were discarded. All parameters were analyzed in all samples together in a single batch at the termination of the experimental protocol (control and patient samples were analysed in the same batch).

### Measurement of serum fetuin-A and RANKL levels

Serum fetuin-A and RANKL levels of patients with BC and of the control group were measured in the venous blood. A commercial kit (Assaypro, USA, cat no: EG 63501-1), based on a quantitative sandwich ELISA, was used and results were determined with ELX 800 UV version ELISA reader and calculated in grams per liter. Mean intra-assay and inter-assay coefficients of variation were less than 4.9% (n:10) and 6.7% (n:10).

### Measurement of other biochemical parameters

Levels of serum glucose, urea, creatinine, total cholesterol, HDL, LDL, AST, ALT, GGT, LDH, ALP, total protein, albumin, parathyroid hormone (PTH), calcium, phosphorus, magnesium, CRP, complete blood count (CBC), CEA, CA 15.3 and erythrocyte sedimentation rate (ESR) were measured in the patient and control groups using the same biochemistry laboratory in our hospital.

### Statistical analysis

Statistical analyses (Mann–Whitney U-test, Student t test) were performed with SPSS 19 (Statistical Package for Social Sciences). The difference in various parameters were analyzed by the Chi-square test. Pearson correlation test was used for correlating fetuin-A, RANKL and the different biochemical parameters. Multivariate logistic regression model was performed to determine the effect of independent risk factors for BC. P-values < 0.05 were considered significant.

## Results

Median age of the patients was 53 (range: 57–86) while it was 47 (range: 23–74) in the healthy group. Patients were 56% postmenopausal, 40% premenopausal and 4% perimenopausal. Twenty-four (20.5%) of the patients had cerbb2 3 positive or cerb2 2 positive and SISH/FISH positive disease. Grade 2 disease was found in 60 (52.6%) patients and 47 patients (41.2%) had grade 3 disease. Seventy-four (63.2%) patients had invasive ductal carcinoma (IDC), 15 (12.8%) had invasive lobular carcinoma (ILC), 6 (5.1%) had mixed (IDC+ILC) carcinoma, 1 had metaplastic cancer and 21 (17.9%) had other favorable (tubular, apocrine, papillary and mucinous) types. Twenty-nine patients (24.7%) had stage I, thirty (25.6%) had stage II and fifty-eight (49.5%) patients had stage III BC.

There was no statistically significant difference between ER status, PR status, cerbb2 status, grade, lymphovascular invasion, perineural invasion, stage, menopausal status and serum parameters. Patients had lower high-density lipoprotein levels (p=0.002) and higher neutrophil counts (p=0.014) rather than the control group (Table 1 and Table 2). Table 3 Fetuin-A and RANKL levels did not differ between the patients and control groups (p=0.116 and p=0.439, respectively) (Table 4). There was no statistically significant difference in fetuin-A levels according to various clinical/laboratory parameters (Table 5). However, patients with favorable histopathologies such as tubular, apocrine, papillary and mucinous subtypes (n=24) had lower RANKL values and it was found to be significant (p=0.04) (Table 6).

**Table 1:** Laboratory values, cell adhesion markers and age of the patients’ and control group.

Age		N	Mean	Std. Dev.	Min	Max	p	units
	Control	37	47.65	11.106	23	74	.014	
	BC	117	53.21	12.026	27	86		
	**Total**	**154**	**51.88**	**12.015**	**23**	**86**		
Fetuin-A (g/L)	Control	37	.35137	.096804	.215	.674	.116	g/L
	BC	117	.38076	.099044	.224	.743		
	**Total**	**154**	**.37370**	**.099000**	**.215**	**.743**		
RANKL (pM)	Control	37	382.895	326.0093	82.9	1763.7	.439	pM
	BC	117	447.340	470.5775	61.1	2486.6		
	**Total**	**154**	**431.856**	**440.0707**	**61.1**	**2486.6**		
Glucose (mmol/L)	Control	37	5.8492	0.7293	4.2185	7.7154	.324	mmol/L
	BC	117	6.2067	2.1565	3.8854	21.7584		
	**Total**	**154**	**6.1207**	1.9169	**3.8854**	**21.7584**		
Urea (mmol/L)	Control	37	23.08	9.8388	8.1585	49.1175	.247	mmol/L
	BC	117	26.1372	14.9833	6.4935	83.4166		
	**Total**	**154**	**25.4029**	**13.9537**	**6.4935**	**83.4166**		
Creatinine (mmol/L)	Control	37	55.83445	11.2394	39.7808	84.8656	.279	mmol/L
	BC	117	58.354	12.5857	34.4767	101.662		
	**Total**	**154**	**57.744**	**12.287**	**34.4767**	**101.662**		

**Table 2:** Laboratory values, cell adhesion markers and age of the patients’ and control group.

Total cholesterol<br>(mmol/L)	Control	37	5.4354	0.9677	3.3364	8.1469	0.814	mmol/L
	BC	117	5.3865	1.1391	3.1553	8.7418		
	**Total**	**154**	**5.3984**	**1.0975**	**3.1553**	**8.7418**		
HDL-Chol (mmol/L)	Control	37	1.475	0.341	0.7242	2.276	0.002	mmol/L
	BC	117	1.2784	0.3279	0.6466	2.1208		
	**Total**	**154**	**1.3255**	**0.3416**	**0.6466**	**2.276**		
LDL-Chol (mmol/L)	Control	37	3.2435	0.7428	1.7587	5.0071	0.654	mmol/L
	BC	117	3.3232	0.9931	0.6052	6.6727		
	**Total**	**154**	**3.3041**	**0.9374**	**0.6052**	**6.6727**		
AST (U/L)	Control	37	21.78	6.872	14	54	0.268	U/L
	BC	117	20.58	5.339	11	43		
	**Total**	**154**	**20.87**	**5.744**	**11**	**54**		
ALT (U/L)	Control	37	21.24	15.673	9	87	0.269	U/L
	BC	117	19.02	8.484	5	56		
	**Total**	**154**	**19.55**	**10.643**	**5**	**87**		
GGT (U/L)	Control	37	21.03	14.544	8	84	0.403	U/L
	BC	117	23.53	16.180	6	104		
	**Total**	**154**	**22.93**	**15.793**	**6**	**104**		
LDH (U/L)	Control	37	178.59	33.815	80	263	0.773	U/L
	BC	117	180.79	42.218	20	296		
	**Total**	**154**	**180.27**	**40.265**	**20**	**296**		
ALP (U/L)	Control	36	78.08	23.285	40	143	0.553	U/L
	BC	116	80.79	24.051	40	205		
	**Total**	**152**	**80.15**	**23.823**	**40**	**205**		
Total protein (g/L)	Control	37	74.435	6.4162	68.0	107.0	0.059	g/L
	BC	116	72.614	4.5792	60.7	81.6		
	**Total**	**153**	**73.054**	**5.1213**	**60.7**	**107.0**		
Albumin (g/L)	Control	37	44.089	2.2737	37.5	48.1	0.463	g/L
	BC	117	43.708	2.8838	35.8	52.3		
	**Total**	**154**	**43.799**	**2.7474**	**35.8**	**52.3**		
Calcium (mmol/L)	Control	37	2.3851	0.0822	2.2456	2.5201	0.201	mmol/L
	BC	117	2.3582	0.1191	1.8963	2.8195		
	**Total**	**154**	**2.3646**	**0.1117**	**1.8963**	**2.8195**		
Phosphorus (mmol/L)	Control	37	1.0448	0.1542	0.7129	1.3419	.994	mmol/L
	BC	116	1.0445	0.1788	0.6161	1.5839		
	**Total**	**153**	**1.0446**	**0.1726**	**0.6161**	**1.5839**		
Magnesium (mmol/L)	Control	37	0.7791	0.0669	0.6213	0.9298	0.232	mmol/L
	BC	116	0.7951	0.0718	0.5842	0.9545		
	**Total**	**153**	**0.7912**	**0.0707**	**0.5842**	**0.9545**		

**Table 3:** Laboratory values, cell adhesion markers and age of the patients’ and control group.

PTH (ng/L)	Control	37	58.981	24.8303	22.8	121.9	0.571	ng/L
	BC	117	62.193	31.3949	11.9	213.5		
	**Total**	**154**	**61.421**	**29.9040**	**11.9**	**213.5**		
Sedimentation<br>(mm/h)	Control	37	22.84	14.299	1	72	0.914	mm/h
	BC	110	23.11	12.883	4	76		
	**Total**	**147**	**23.04**	**13.204**	**1**	**76**		
CRP (nmol/L)	Control	37	36.7514	36.1676	4.9524	148.9524	0.319	nmol/L
	BC	117	47.3524	61.0316	2.0952	434.2857		
	**Total**	**154**	**44.7924**	**56.1467**	**2.0952**	**434.2857**		
WBC (10^9^/L)	Control	37	6.7614	1.10867	4.43	9.45	0.086	10^9^/L
	BC	117	7.3162	1.84842	3.63	13.12		
	**Total**	**154**	**7.1829**	**1.71353**	**3.63**	**13.12**		
Neutrophil<br>(10^9^/L)	Control	37	3.9443	1.00572	1.90	5.82	0.014	10^9^/L
	BC	117	4.6630	1.66258	2.00	10.53		
	**Total**	**154**	**4.4903**	**1.55839**	**1.90**	**10.53**		
Lymphocyte<br>(10^9^/L)	Control	37	2.1519	.50326	1.25	3.41	0.328	10^9^/L
	BC	117	2.0429	.61292	1.05	4.41		
	**Total**	**154**	**2.0691**	**.58873**	**1.05**	**4.41**		
PLT<br>(10^9^/L)	Control	37	264.32	49.311	160	356	0.895	10^9^/L
	BC	117	265.88	65.808	123	524		
	**Total**	**154**	**265.51**	**62.097**	**123**	**524**		
MPV (Fl)	Control	37	10.408	.8958	8.7	13.8	0.708	Fl
	BC	115	10.481	1.0618	8.4	14.2		
	**Total**	**152**	**10.463**	**1.0215**	**8.4**	**14.2**		
Ca 15.3<br>(U/mL)	Control	5	15.940	5.6145	10.7	24.1	0.733	U/mL
	BC	104	22.993	45.8327	3.0	392.6		
	**Total**	**109**	**22.670**	**44.7968**	**3.0**	**392.6**		
CEA(μg/L)	Control	4	3.117	4.6920	.3	10.1	0.854	mg/L
	BC	103	3.864	8.0352	.3	56.4		
	**Total**	**107**	**3.836**	**7.9228**	**.3**	**56.4**		

**Table 4:** Serum fetuin-A and RANKL levels in the patients’ and the control group.

	N	Mean	Standard deviation	Minimum	Maximum	p
Fetuin-A<br>(g/L)	Control 37<br>BC 117<br>**Total 154**	0.35137<br>0.38076<br>**0.37370**	0.096804<br>0.099044<br>**0.99000**	0.215<br>0.224<br>**0.215**	0.674<br>0.743<br>**0.743**	<br>0.116
RANKL<br>(pM)	Control 37<br>BC 117<br>**Total 154**	382.895<br>447.340<br>**431.856**	326.0093<br>470.5775<br>**440.0707**	82.9<br>61.1<br>**61.1**	1763.7<br>2486.6<br>**2486.6**	<br>0.439

**Table 5:** Comparisons of fetuin-A levels according to various clinical/laboratory parameters. Significant p values (less than 0.05) are highlighted in bold.^*^ Apocrine, papillary, mucinous, metaplastic, neuroendocrine, tubular. ^** ^Invasive ductal carcinoma, invasive lobular carcinoma, mixed type.

Variables	Fetuin Median (Range)	n	P
Ki-67			
≤ 30	0.345 (0.235–0.522)	43<br>71	0.09
> 30	0.389 (0.224–0.743)	71	
ER			
Negative	0.398 (0.264–0.692)	25	0.38
Positive	0.375 (0.224–0.743)	92	
PR			
Negative	0.399 (0.247–0.692)	30	0.38
Positive	0.373 (0.224–0.743)	87	
HER2			
Negative	0.362 (0.224–0.743)	90	0.71
Positive	0.369 (0.249–0.692)	24	
Classification			
Luminal	0.358 (0.224–0.743)	92	0.39
Others	0.392 (0.264–0.692)<br>9225	25	
Classification			
Triple positive	0.345 (0.249–0.497)	13	0.40
Others	0.368 (0.224–0.743)	100	
Classification			
Triple negative	0.336 (0.267–0.591)	11	0.63
Others	0.362 (0.224–0.743)	106	
Classification			
HER2 enriched	0.369 (0.249–0.692)	24	0.71
Others	0.362 (0.224–0.743)	90	
Histological subgroups			
Favorable groups^*^	0.355 (0.242–0.526)<br>0.355 (0.242-0.526)	24	0.51
Unfavorable groups^**^	0.361 (0.224–0.743)	93	
Stage			
Early stage	0.360 (0.224–0.650)	60	0.55
Local advanced stage	0.370 (0.235–0.743)	54	

**Table 6:** Comparisons of RANKL levels according to various clinical/laboratory parameters. Significant p values (less than 0.05) are highlighted in bold. ^*^ Apocrine, papillary, mucinous, metaplastic, neuroendocrine, tubular. ^**^ Invasive ductal carcinoma, invasive lobular carcinoma, mixed type.

Variables	RANKL Median (Range)	n	P
Ki-67			
≤ 30	203.300 (61.100–1577.500)	43	0.16
> 30	273.300 (101.800–2486.600)	71	
ER			
Negative	490.888 (101.800–2486.600)	25	0.54
Positive	434.228 (61.100–1890.500)	92	
PR			
Negative	441.337 (101.800–2486.600)	30	0.66
Positive	448.059 (61.100–1890.500)	87	
HER2			
Negative	240.950 (61.100–1973.600)	90	0.61
Positive	276.600 (101.800–2486.600)	24	
Classification			
Luminal	250.250 (61.100–1890.500)	92	0.54
Others	294.500 (101.800–2486.600)	25	
Classification			
Triple positive	314.600 (121.700–956.700)	13100	0.22
Others	239.250 (61.100–2486.600)		
Classification			
Triple negative	372.900 (113.200–1973.600)	11	0.50
Others	250.250 (61.100–2486.600)	106	
Classification			
HER2 enriched	276.600 (101.800–2486.600)	24	0.61
Others	240.950 (61.100–1973.600)	90	
Histological subgroups			
Favorable groups^*^	184.500 (80.500–638.300)	24	0.04
Unfavorable groups^**^	269.100 (61.100-2486.600)	93	
Stage			
I and II	254.600 (61.100–1973.600)	60	0.62
III	261.450 (101.800–2486.600)	54	

## Discussion

For BC, different serum markers were evaluated up to now and some of them are found to be prognostic, some are diagnostic and/or predictive [11]. Fetuin-A was originally discovered to be an inhibitor of vascular calcification. Furthermore it is demonstrated that it plays an important role in free fatty acid induced insulin resistance in the liver [12] [13]. Increased fe tuin-A had been also been linked to increased occurrence of non-alcoholic fatty liver disease and cardiovascular events, believed to be due to its pro inflammatory effects. Thus, in contrast it has some anti-inflammatory properties. It is a negative acute-phase reactant in sepsis, promotes wound healing, and is neuroprotective [14]. The potential role of fetuin-A in tumor progression stemmed from earlier studies that suggested that it was the cell attachment factor in serum [15]. In head and neck squamous cell carcinoma (HNSCC), there was an increased expression of a higher molecular weight fetuin-A [16]. There is ectopic synthesis of fetuin-A by divergent cancer cell lines [17]. Patients with high ectopic expression of fetuin-A in lung cancer and gastric cancer tend to have lower survival [18] [19]. Fetuin-A is an important marker in the tumor microenvironment, for cancer stem cells and for matrix metalloproteinases [20] [21].

Fetuin-A is found to be a serum biomarker for colorectal cancer patients [22]. It is found to be increased in malignant pleural effusion of lung cancer patients [23]. Furthermore, in a study done in Mexican BC population, the presence of serum autoantibodies against fetuin-A protein found to be useful as serum biomarkers for early-stage BC screening [9]. Fetuin-A seems to be a serum chemo-attractant protein that also promotes invasion of BC tumor cells [24].

In our study, we found no association of serum fetuin-A levels for BC patients with other laboratory parameters and with control subjects. This may be a result of exploring only early staged patients. In the Mexican BC population [9], there were 36 patients (30 with ductal and 6 lobular carcinoma) but they used an immune proteomic approach, combining two-dimensional (2D) electrophoresis, Western blot, and matrix-associated laser desorption/ionization mass spectrometry (MALDI-MS) methods. We used one method which was the ELISA method for the detection of fetuin-A levels. We performed this study in 117 patients with invasive ductal, lobular, tubular, papillary and mucinous cancers. However, in our study, there was a trend to be lower for fetuin-A levels for more favorable histologic subtypes. It is very well known that BC has different histologic subtypes as well as its diffrenet molecular characteristics. In the Mexican study [9], there is no data about the tumors’ molecular characteristics such as ER, PR and cerbb2 status. In our study; there are older patients than the other study. Taken together all these discrepancies, in our study which was done in Turkish BC patients, fetuin-A levels did not differ.

RANKL/RANK system is seen as a downstream mediator of progesterone-driven mammary epithelial cells proliferation, BC initiation and progression. Expression of RANKL, RANK has been detected in BC cell lines and in human primary BCs. To date, dysregulation of RANKL/RANK at the skeletal level has been widely documented in the context of metastatic bone disease [25]. The interference with the RANK/RANKL system could therefore serve as a potential target for prevention and treatment of BC [26] [27]. For metastatic BC patients, specifically for patients with bone metastasis, RANKL levels were found to be diagnostic and somewhat predictive for therapy [26]. In our study; for early staged BC patients, RANKL levels were found to be lower in the favorable histological subtypes of BC. This is a new topic for early stage BC patients.

## Conclusion

We found a correlation between serum RANKL levels and favorable histological subtypes of BC.However, there was no significance between fetuin-A levels and other clinical/laboratory parameters. Further and detailed studies can enlighten the role of these cell adhesion markers better for BC patients.

## Dodatak

### Conflict of interest statement

All the authors declare that they have no conflict of interest in this work.
